# Signaling regulation and role of filamin A cleavage in Ca^2+^-stimulated migration of androgen receptor-deficient prostate cancer cells

**DOI:** 10.18632/oncotarget.9472

**Published:** 2016-05-19

**Authors:** Chunfa Huang, R. Tyler Miller, Carl E. Freter

**Affiliations:** ^1^ Division of Hematology and Oncology, Department of Internal Medicine, School of Medicine, Saint Louis University, Saint Louis, MO 63104, USA; ^2^ Departments of Medicine and Dallas VAMC, University of Texas Southwestern Medical Center, Dallas, Texas 75390, USA

**Keywords:** Ca^2+^-sensing receptor, filamin a cleavage, calpain, p115RhoGEF, AR-deficient prostate cancer cells

## Abstract

Ca^2+^, a ubiquitous cellular signal, and filamin A, an actin-binding protein, play an important role in the regulation of cell adhesion, shape and motility. Using transwell filters to analyze cell migration, we found that extracellular Ca^2+^ (Ca_o_^2+^) promotes the migration of androgen receptor (AR)-deficient and highly metastatic prostate cancer cell lines (DU145 and PC-3) compared to AR-positive and relatively less metastatic prostate cancer cells (LNCaP). Furthermore, we found that expression of filamin A is up-regulated in DU145 and PC-3 cells, and that Ca_o_^2+^ significantly induces the cleavage of filamin A. Silencing expression of Ca^2+^-sensing receptor (CaR) and p115RhoGEF, and treating with leupeptin, a protease inhibitor, and ALLM, a calpain specific inhibitor, we further demonstrate that Ca_o_^2+^-induced filamin A cleavage occurs via a CaR- p115RhoGEF-calpain dependent pathway. Our data show that Ca_o_^2+^ via CaR- mediated signaling induces filamin A cleavage and promotes the migration in AR-deficient and highly metastatic prostate cancer cells.

## INTRODUCTION

Prostate cancer is the most commonly diagnosed cancer and the second leading cause of cancer deaths among men in the United States and many developed countries [1, 2]. Metastasis of prostate cancer cells is associated with increased mortality and reduced treatment effectiveness [3]. The process of metastasis involves a series of sequential steps, which include neoangiogenesis and lymphangiogenesis, loss of adhesion with migration away from the primary tumor and entry into the systemic vasculature or lymphatics. Metastatic growth in sites such as lymph nodes and bone marrow represents the specific non-random homing of prostate cancer cells [4]. Understanding the molecular mechanism of cancer metastasis and targeting the key signaling molecules involved in this process could lead to new therapeutic drug discovery with new mechanism.

Cell migration requires controlled modification of the actin cytoskeleton, specifically the polymerization of actin filaments underneath the plasma membrane. Actin forms filaments that provide cells with mechanical support and the driving force for movement [5, 6]. Numerous physiological and pathological stimuli promote the reorganization of actin cytoskeleton, thereby modulating cell migration and cancer metastasis. Filamin, an actin-binding protein that was discovered almost 40 years ago, organizes cortical actin filaments and dynamic three-dimensional networks in the leading edges of migrating cells [7–9]. Filamin as a scaffold protein also interacts with various membrane proteins and intracellular mediators of adhesion and migration, and serves as a mechanical element in membrane ruffle formation [10, 11]. Earlier studies indicated that filamin A interacts with androgen receptor (AR) in a yeast two hybrid interaction trap assay and also facilitates AR nuclear translocation and movement [12]. Some studies later showed that filamin A can be cleaved into two fragments (~100 kD and 180 kD) in prostate cancer cells, HeLa cells and A7 melanoma cells [13, 14]. A C-terminal 100-kDa fragment of filamin A co-localizes with AR to the nucleus, and represses AR transactivation [14]. By immunohistochemistry, Bedolla et al. found that the small fragment of filamin A has nuclear localization in benign prostate and cytoplasmic localization in metastatic prostate cancers [15]. Increasing nuclear filamin A fragment induces prostate cancer cell apoptosis during androgen deprivation therapy (ADT) [16]. Androgen-triggered AR association with filamin A and integrin β1 leads to activation of focal adhesion kinase (FAK) and migration in NIH3T3 cells [17]. These studies indicate that the interaction of AR and filamin A fragment plays an important role in AR-sensitive prostate cancer cell migration and invasion. However, the signaling pathway that regulates filamin A cleavage and cell migration in AR- deficient prostate cancer cells has not been studied.

Ca^2+^ is a ubiquitous cellular signal which plays a central role in the regulation of cell growth, proliferation, differentiation, migration and apoptosis [18, 19]. Increased calcium intake from dairy products has been suggested as a risk factor for prostate cancer [20–22]. Extracellular Ca^2+^ (Ca_o_^2+^) as a primary signaling molecule acts through the Ca^2+^-sensing receptor (CaR, a G protein coupled receptor) which directly regulates cell signal transduction and the Ca^2+^ channels which can elevate intracellular Ca^2+^ levels to modulate Ca^2+^-dependent proteins [18, 19]. Using a yeast two hybrid assay, we and others reported that the CaR interacts with filamin A [23, 24], and this interaction participates in CaR-mediated multiple signaling pathways in HEK 293 cells [23–25]. More recently, we found that expression of the CaR is up-regulated in prostate cancer cells and prostate tumor specimens compared to nonmalignant prostate epithelial cells and normal prostate specimens [26]. Liao et al. recently reported that silencing of CaR expression by CaR-specific shRNA reduces PC-3 cell growth rate and suppresses tumor progression [27]. Whether CaR-mediated signaling is involved in filamin A cleavage and regulating migration in AR-deficient and highly metastatic prostate cancer cells is still unknown.

In this study, we compared the expression and cleavage of filamin A in normal prostate and prostate cancer cells, explored how Ca_o_^2+^ regulates the cleavage of filamin A in prostate cancer cells, and determined whether the cleavage of filamin A is associated with prostate cancer cell migration. Our data show that Ca_o_^2+^ via CaR-mediated signaling induces filamin A cleavage and promotes the migration in AR-deficient and highly metastatic prostate cancer cells.

## RESULTS

### Ca_o_^2+^ induces the migration of AR-deficient prostate cancer cells

**Figure 1:**
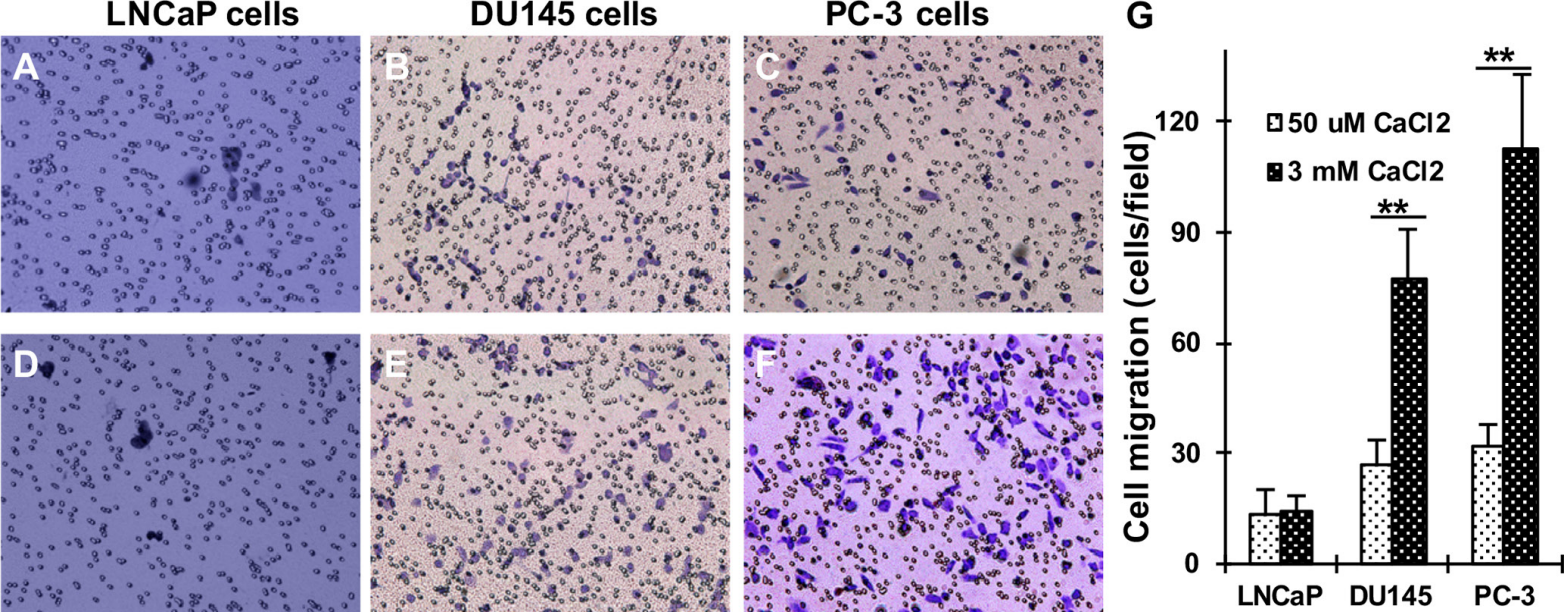
Effect of Ca_o_^2+^ on the migration of LNCaP, DU145 and PC-3 cells LNCaP, DU145 and PC-3 cells were seeded in 24-well plates with Transwell filters and the lower chamber contained 50 μM (**A–C**) or 3 mM (**D–F**) CaCl_2_. The cells were cultured in a 37^°^C incubator for 18 hrs, and the migrating cells were determined. (**G**) The data represent means of forty fields with duplicate samples in two experiments. **P < 0.01.

Recently, we found that the levels of CaR, Gα_12_ and p115RhoGEF expression are significantly up-regulated in AR-deficient and highly metastatic prostate cancer cells and prostate tumor specimens [26]. The activation of CaR-signaling increases in prostate cancer cell attachment [27] and breast cancer cell migration [28]. To explore the role of Ca_o_^2+^ in the regulation of cell migration, LNCaP, DU145 and PC-3 cells were cultured on Transwell filters with lower chambers containing different concentrations of Ca_o_^2+^ to determine whether Ca_o_^2+^ promotes prostate cancer cell migration. With the increases in Ca_o_^2+^ concentration in the lower chamber, we found that DU145 and PC-3, the AR- deficient and highly metastatic prostate cancer cell lines, had increased numbers of migrating cells in the presence of elevated Ca_o_^2+^ (3 mM) versus low Ca_o_^2+^ (50 μM). In contrast, LNCaP cells, an androgen-sensitive and less metastatic prostate cancer cell line, had similar cell numbers of migrating regardless of the Ca_o_^2+^ levels (Figure 1).

### Up-regulation of filamin A expression in AR-deficient and highly metastatic prostate cancer cells

**Figure 2:**
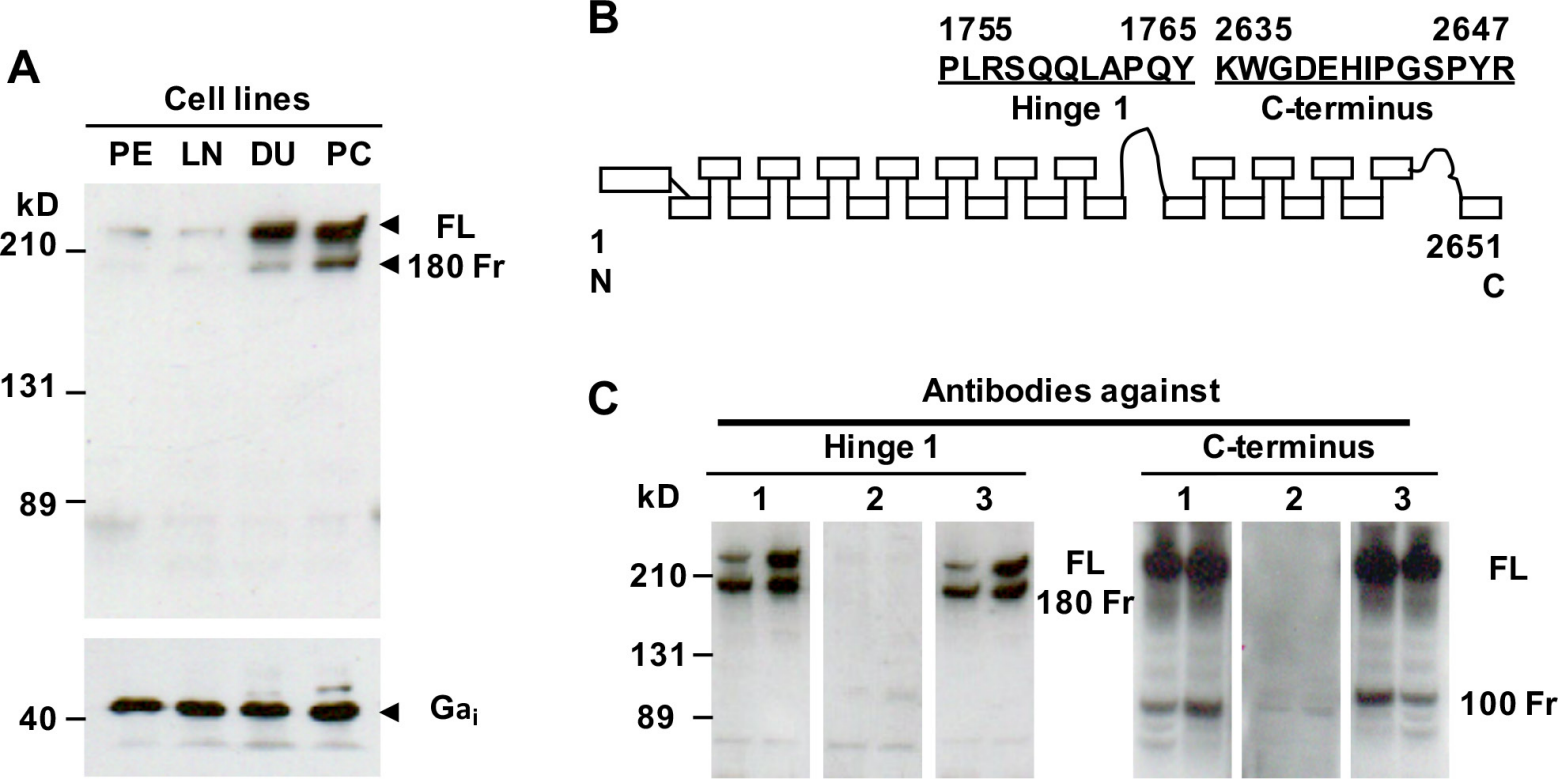
Endogenous filamin A expression and characterization of two anti-filamin A antibodies (**A**) Equal amounts of cellular protein from PE, LNCaP (LN), DU145 (DU) and PC-3 (PC) cells were processed for immunoblotting using the antibodies against filamin A and Gα_i_ as a loading control. (**B**) The peptides used to generate the anti-filamin A antibodies against different domains of human filamin A. (**C**) Characterization of the anti-filamin antibodies. Lysates from DU145 and PC-3 cells were processed for immunoblotting using two polyclonal antibodies which recognize the hinge 1 or C-terminal regions of human filamin A (1). These antibodies were preincubated with the antigenic peptides (2) and a non-specific peptide (3). The data represent three experiments with duplicate samples. FL, full length of filamin A; 180 Fr, 180 kD and 100 Fr, 100kD fragment.

Filamin A can be cleaved to two fragments (~100 kD and 180 kD) in prostate cancer cells [13, 14], and the cleavage of filamin A is associated with prostate cancer metastasis [15]. To investigate whether filamin A plays an important role in prostate cancer metastasis, we first assessed the expression of filamin A in human nonmalignant prostate epithelial cells (PE), LNCaP, DU145 and PC-3 cells. Equal amounts of cellular protein from these four cell lines were processed for immunoblotting using an anti-filamin A antibody which recognizes the hinge 1 region of human filamin A. Two specific bands (280 kD, full length and ~180 kD, a fragment) were identified in these cell lines, but the levels of expression were significantly different. The endogenous filamin A expression was substantially higher in DU145 and PC-3 cell lines than those in PE cells and LNCaP cells (Figure 2A). To assess the cleavage of filamin A, we made two different polyclonal anti-filamin A antibodies. Figure 2B illustrates that the immunogenic peptides that correspond to the hinge 1 and C-terminal amino acid sequences of human filamin A. The specificity of the antibodies was determined by peptide blocking (Figure 2C). Using the anti-filamin A antibody which recognizes the hinge 1 region, we detected two specific bands at ~180 kD and 280 kD, while the antibody that recognizes the C-terminal region detects two specific bands at ~100 kD and 280 kD.

### Ca_o_^2+^ induces the cleavage of filamin A in AR-deficient and highly metastatic prostate cancer cells

**Figure 3:**
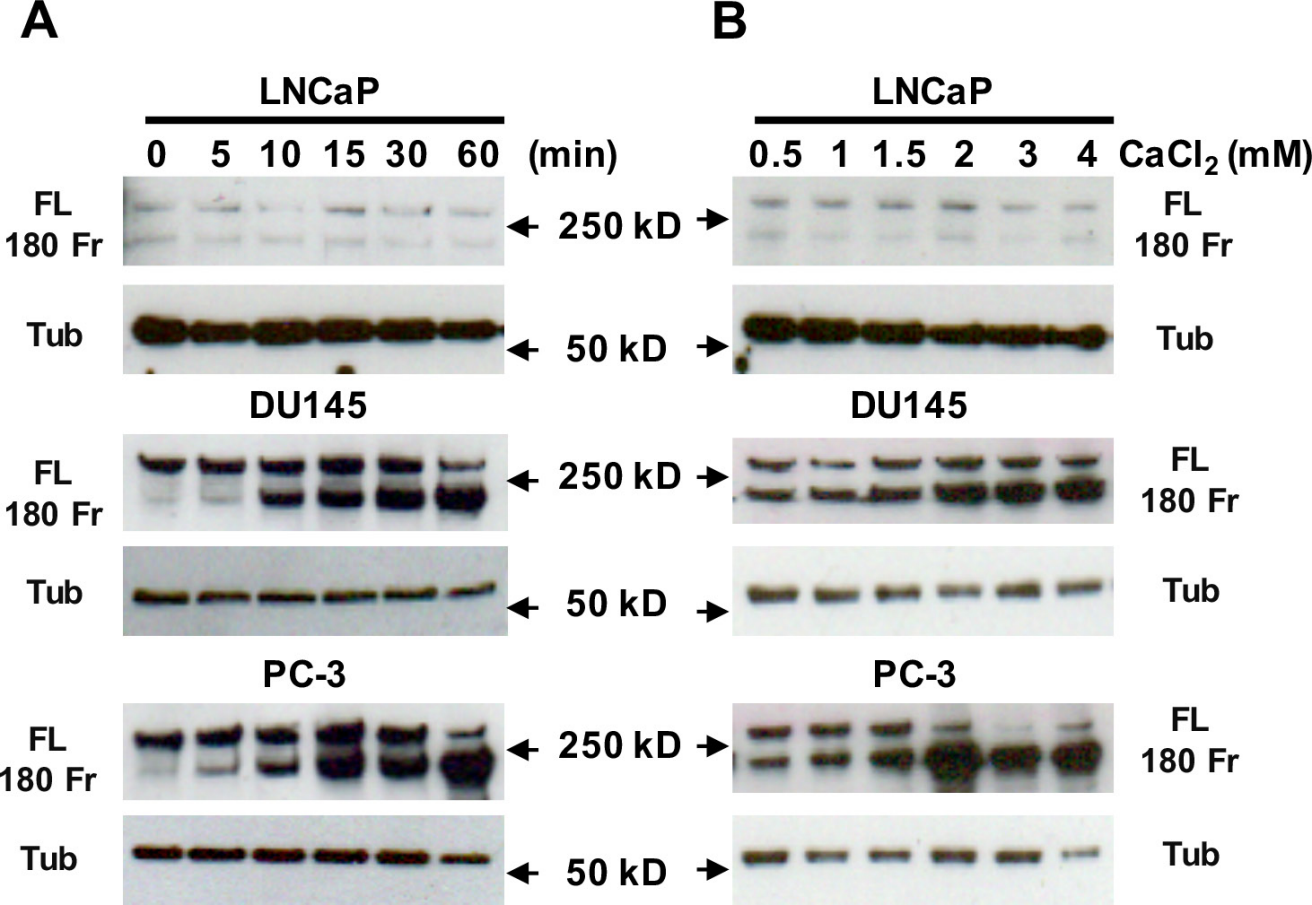
Effect of Ca_o_^2+^ on the cleavage of filamin A LNCaP, DU145 and PC-3 cells were serum-deprived overnight, and then stimulated with 3 mM CaCl_2_ for different periods of time or different concentrations of CaCl_2_ for 1 hr. Equal amounts of cellular protein were processed for immunoblotting using the antibodies against filamin A or tubulin. The data represent three experiments with duplicate samples. FL, full length of filamin A, 180 Fr, 180 kD fragment, and Tub, tubulin.

**Figure 4:**
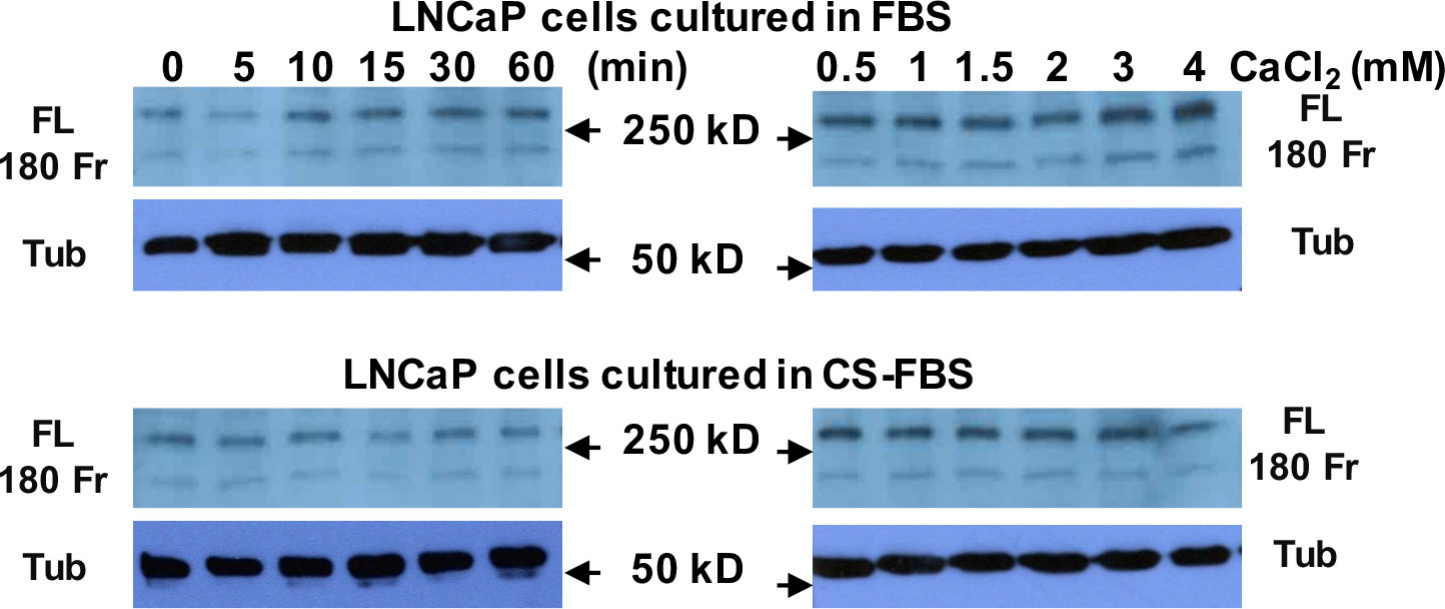
Androgen deprivation does not affect Ca_o_^2+^-induced filamin A cleavage in LNCaP cells LNCaP cells were cultured in either 10% fetal bovine serum (FBS) or 10% charcoal-stripped fetal bovine serum (CS-FBS) for 3 days (up to 75% confluence) and then were serum-deprived overnight. The cells were stimulated with 3 mM CaCl_2_ for different periods of time or different concentrations of CaCl_2_ for 1 hr. Equal amounts of cellular protein were processed for immunoblotting using the antibodies against filamin A or tubulin. FL, full length of filamin A, 180 Fr, 180 kD fragment, and Tub, tubulin.

**Figure 5:**
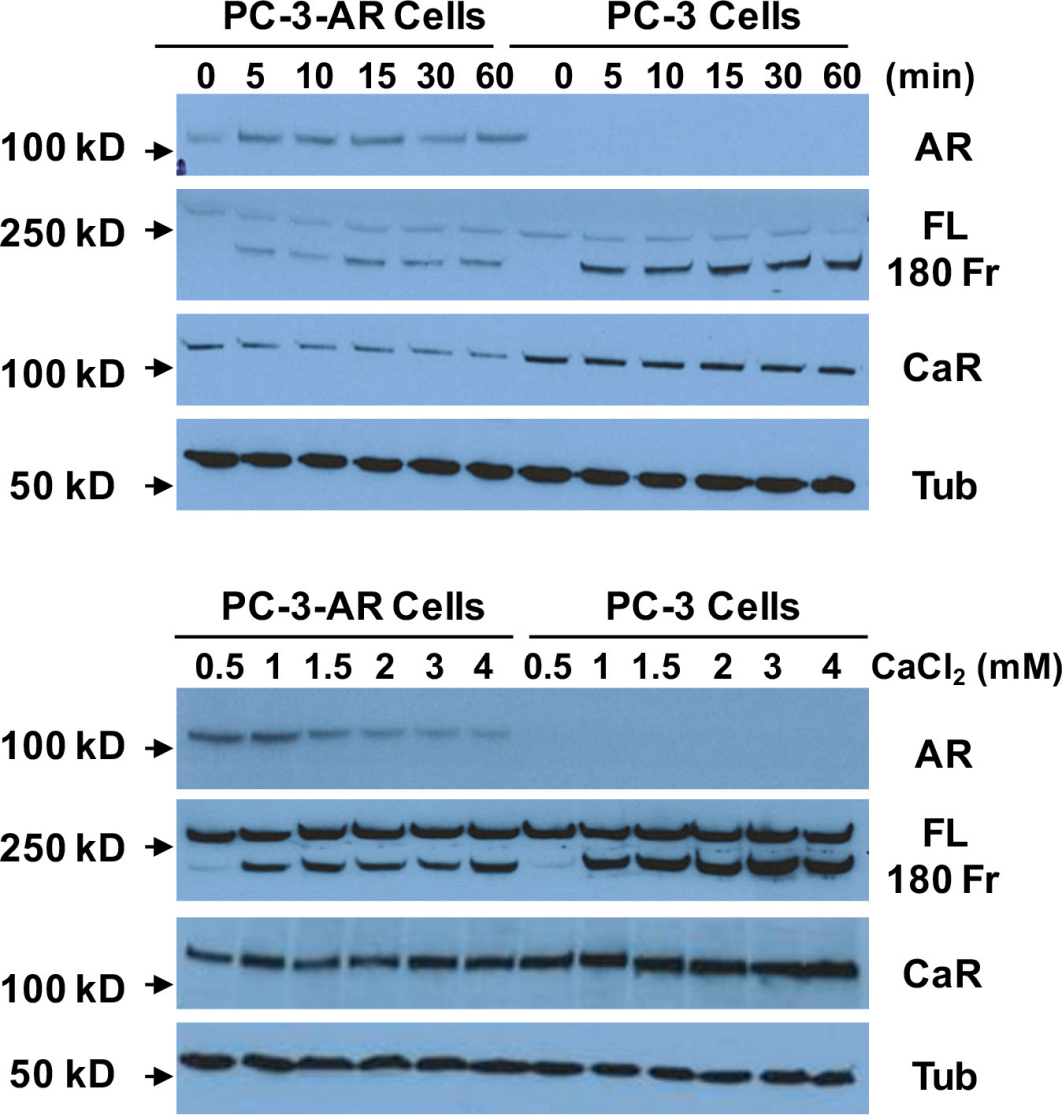
Effect of AR-expression on Ca_o_^2+^-induced filamin A cleavage PC-3 cells and AR-expressing PC-3 cells were cultured for 3 days (up to 75% confluence) and then were serum-deprived overnight. The cells were stimulated with 3 mM CaCl_2_ for different periods of time or different concentrations of CaCl_2_ for 1 hr. Equal amounts of cellular protein were processed for immunoblotting using the antibodies against androgen receptor (AR), filamin A (FL, full length and 180 Fr, 180 kD fragment), Ca^2+^-sensing receptor (CaR) and tubulin (Tub).

To study whether Ca_o_^2+^ induces the cleavage of filamin A in prostate cancer cells, LNCaP, DU145 and PC-3 cells were treated with 3 mM Ca_o_^2+^ for different periods of time, and the samples were analyzed by immunoblotting. The data in Figure 3A show that Ca_o_^2+^ induces time-dependent cleavage of filamin A in DU145 and PC-3 cells, but not in LNCaP cells. Filamin A is cleaved in response to Ca_o_^2+^ beginning at 5 min and increases up to one hour. We also investigated the effect of Ca_o_^2+^ concentration on filamin A cleavage in LNCaP, DU145 and PC-3. Figure 3B illustrates the dose-response of Ca_o_^2+^-induced cleavage of filamin A in DU145 and PC-3 cells. This dose-dependent filamin A cleavage reached a plateau at approximately 2 mM Ca_o_^2+^. Again, LNCaP cells did not respond to Ca_o_^2+^-stimulation. To test whether androgen modulates Ca_o_^2+^-induced filamin A cleavage in LNCaP cells, the cells were cultured in media containing either 10% fetal bovine serum or 10% charcoal-stripped fetal bovine serum, and then treated with Ca_o_^2+^. Figure 4 shows that charcoal-stripped androgen does not affect Ca^2+^-induced filamin A cleavage in LNCaP cells. We also compared the cleavage of filamin A in PC-3 cells and AR-expressing PC-3 cells, and found that AR expression in PC-3 cells interferes with filamin A cleavage by reducing the expression of CaR and filamin A and the cleavage of filamin A (Figure 5). These data demonstrate that Ca_o_^2+^ induces AR-independent filamin A cleavage and enhances filamin A cleavage in AR-deficient and highly metastatic prostate cancer cells.

### Ca_o_^2+^-induced filamin A cleavage is CaR-dependent

**Figure 6:**
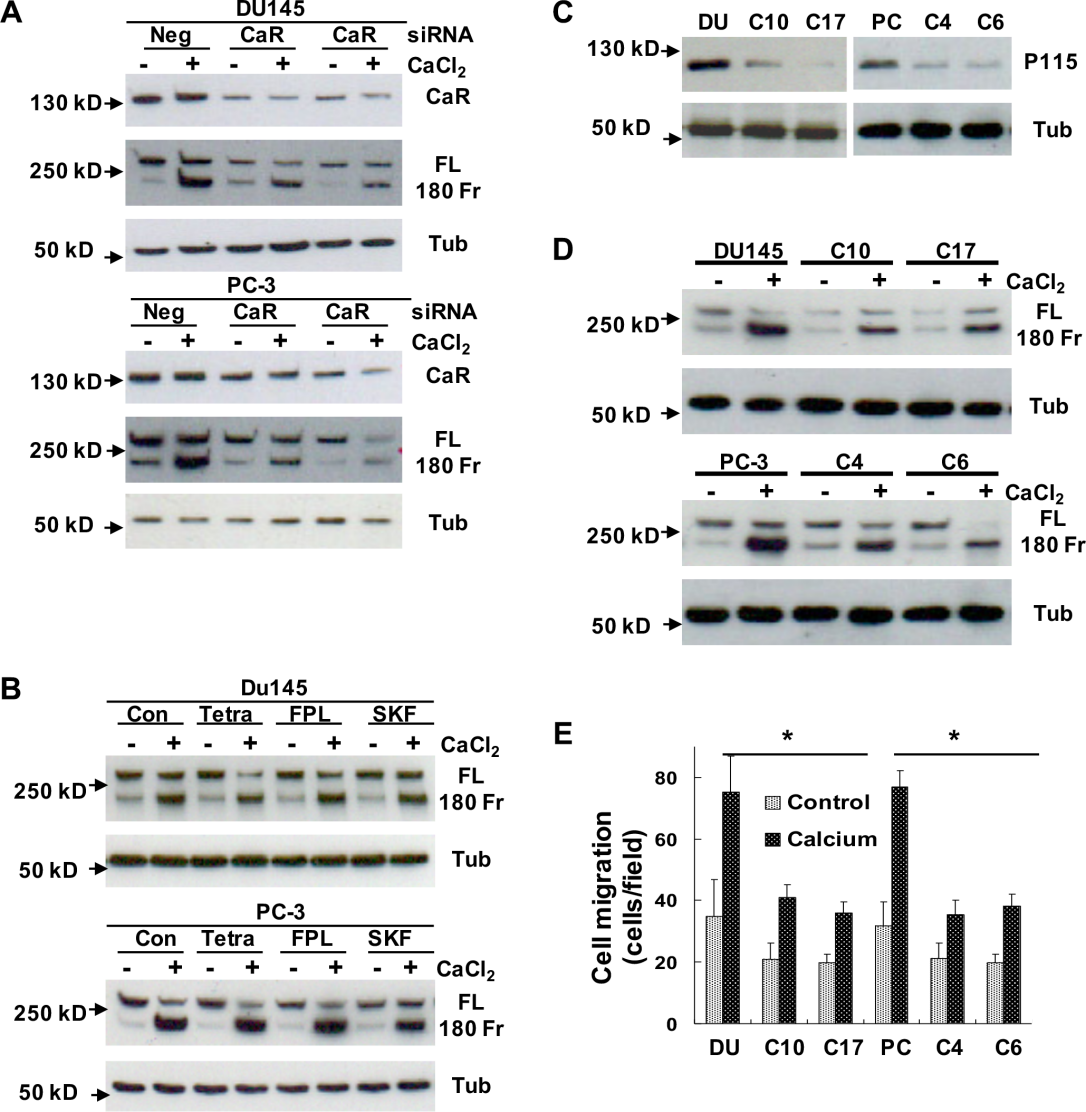
Determination of the role of CaR-mediated signaling in the cleavage of filamin A (**A**) DU145 and PC-3 cells were transfected with negative control (Neg) or CaR-specific siRNA (CaR), (**B**) DU145 and PC-3 cells were serum-deprived overnight last hour with different chemical regulators, (**C** and **D**) DU145 or PC-3 cells as well as their stable variants with silenced p115RhoGEF expression were serum deprived overnight. The serum deprived cells were treated with or without 3 mM CaCl_2_ for 60 mins, and then the samples were processed for immunoblotting using the antibodies against CaR, p115RhoGEF, filamin A or tubulin. (**E**) DU145, PC3 and their stable variants were seeded in 24-well plates with Transwell filters, and then analyzed for Ca_o_^2+^-induced cell migration. The data represent means of 40 fields with duplicate samples in two experiments. *P < 0.05. Con, control; CaR, Ca^2+^-sensing receptor; FL, full length of filamin A; 180 Fr, 180 kD fragment of filamin A; Tub, tubulin; Tetra, tetrandrine: FPL, FPL-64176; SKF, SK&F96365; and p115, p115RhoGEF. The clonal cells with stably silenced p115RhoGEF are from DU145 (C10 and C17) and PC-3 (C4 and C6) cells.

Ca_o_^2+^ can act through the CaR that directly regulates cell signal transduction or the Ca^2+^ channels that elevate intracellular Ca^2+^ levels to modulate Ca^2+^-dependent proteins [18, 19]. To investigate whether the CaR or/and Ca^2+^ channels play a role in Ca_o_^2+^-induced filamin A cleavage, we analyzed the cleavage of filamin A in DU145 and PC-3 cells that were transfected with CaR-specific siRNA or pretreated with Ca^2+^-channel regulators. As shown in Figure 6A, transfection of CaR-specific siRNA into DU145 and PC-3 cells significantly reduced CaR expression, and also attenuated the cleavage of filamin A. However, pretreatment with Ca^2+^-channel regulators, such as tetrandrine (a Ca2+-channel blocker), FPL-64176 (a Ca^2+^-channel activator), and SK&F96365 (a Ca^2+^ entry inhibitor), did not significantly affect the cleavage of filamin A in response to Ca_o_^2+^-stimulation (Figure 6B). Expression of the CaR, Gα12 and p115RhoGEF are significantly up-regulated in DU145 and PC-3 cells and prostate tumor specimens [26, 29]. CaR-Gα12-p115RhoGEF signaling stimulates choline kinase activity in AR-deficient and highly metastatic prostate cancer cells [26]. To determine the role of CaR-Gα12-p115RhoGEF signaling in the cleavage of filamin A, we established DU145 and PC-3 cell lines that stably silence p115RhoGEF [26], and analyzed the effect of p115RhoGEF on Ca_o_^2+^-induced filamin A cleavage. Figures 6C, 6D and 6E clearly show that silencing of p115RhoGEF not only reduces the cleavage of filamin A, but also reduces DU145 and PC-3 cell migration in response to Ca_o_^2+^. These data indicate that Ca_o_^2+^initially binds to the CaR and activates CaR-mediated signaling to regulate the cleavage of filamin A in AR-deficient and highly metastatic prostate cancer cells.

### Ca_o_^2+^ induces the cleavage of filamin A and cell migration via calpain

**Figure 7:**
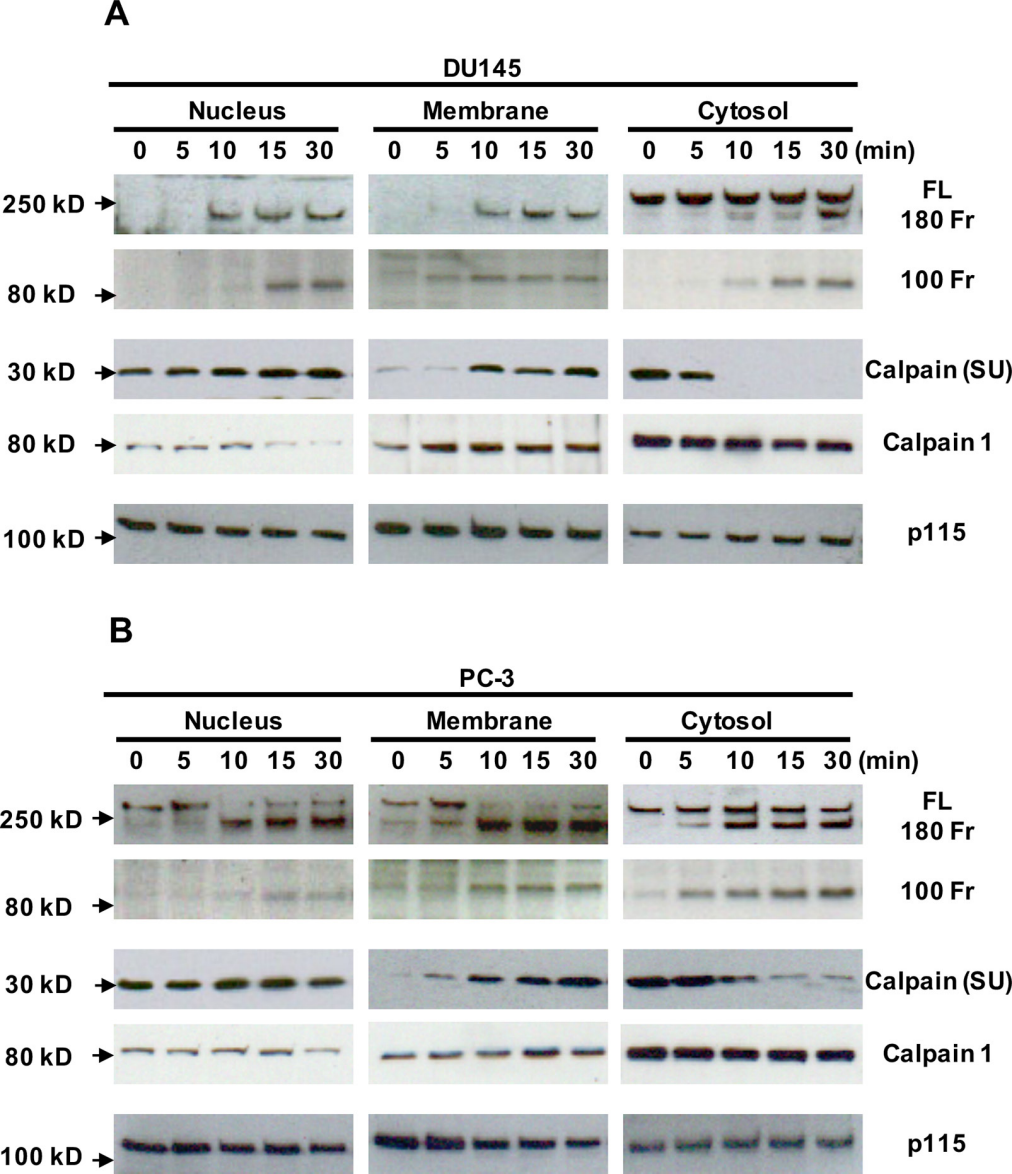
Translocation of calpain and filamin A fragments in Ca_o_^2+^-treated DU145 and PC-3 cells by cellular fractionation DU145 (**A**) and PC-3 (**B**) cells were cultured in 100 mm dishes, serum deprived overnight, and then stimulated with 3 mM Ca_o_^2+^ for 0, 5, 10 15 or 30 min. The cells in the dishes were harvested and fractionated to yield nuclei, crude membranes and cytosol. Equal amounts of cellular proteins from different fractions were processed for immunoblotting using the antibodies against the first hinge region and the C-terminus of filamin A (FL, full length; 180 Fr, 180 kD fragment and 100 Fr, 100 kD fragment), the small unit of calpain 1 and 2 [calpain (SU)], calpain 1 and p115RhoGEF (p115). The data are representative of three experiments with duplicate samples.

**Figure 8:**
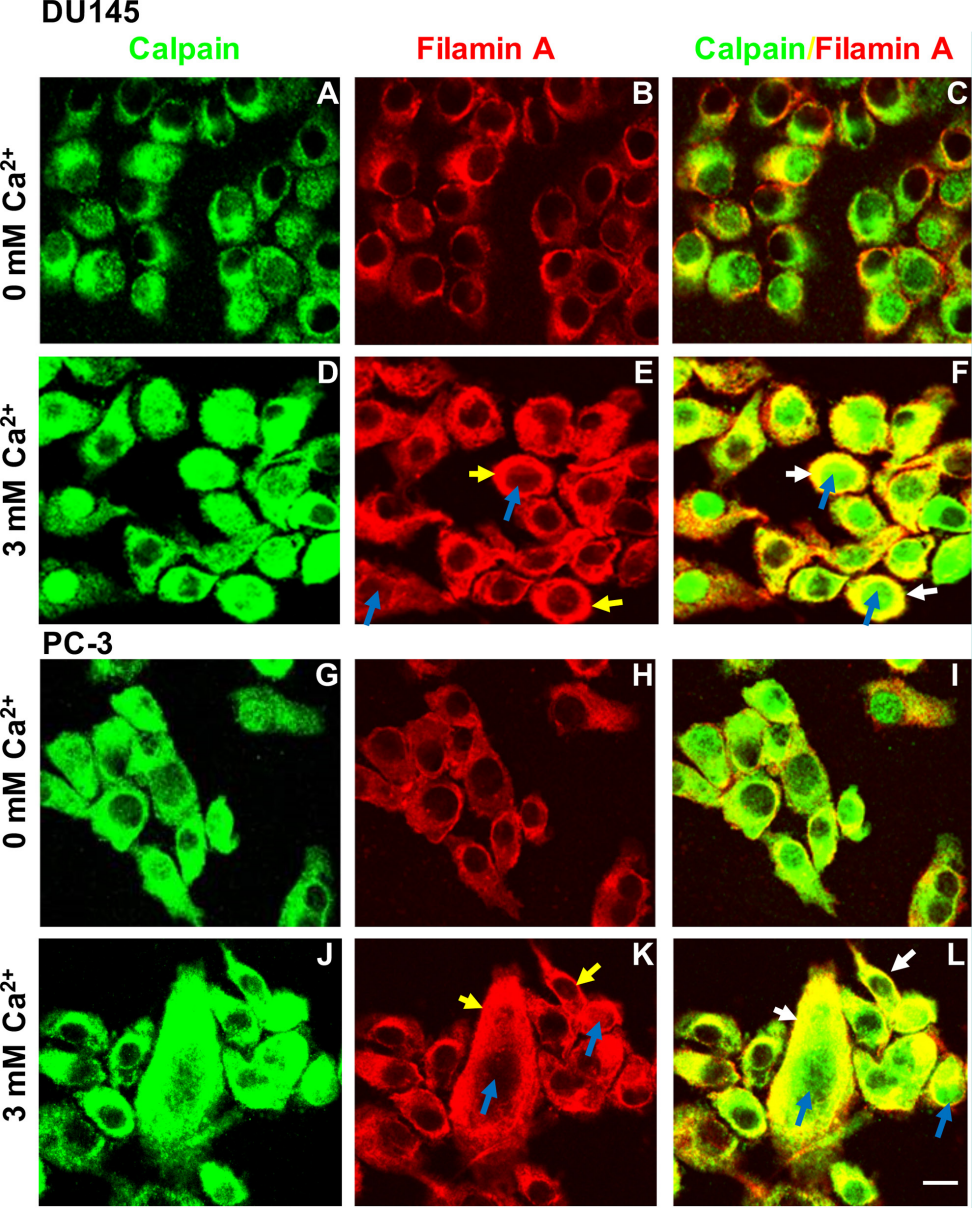
Immunocytochemistry of Ca_o_^2+^-induced translocation of calpain and filamin A in DU145 and PC-3 cells DU145 (**A**–**F**) and PC-3 (**G**–**L**) cells were cultured in 100 mm dishes with two coverslips per dish, serum deprived overnight, and then stimulated without (A–C and G–I) or with 3 mM Ca_o_^2+^ for 30 min (D–F and J–L). The cover slips were processed for immunocytochemistry using the polyclonal anti-filamin A and monoclonal anti-calpain (small unit) antibodies, and appropriate secondary antibody. Fluorescent confocal micrographs are representative of several experiments with duplicate coverslips. Ca_o_^2+^-induced translocation of calpain and filamin A shows in the nuclei (blue arrows) or plasma membrane (yellow arrows). White arrows show the co-localization of filamin A and calpain in the plasma membrane. Scale bar = 15 μm.

It has been shown that filamin A can be cleaved through activation of calcium-activated proteases, such as calpains [7, 30–32], and that the filamin A fragments can be redistributed in cells [15, 30]. To investigate whether Ca_o_^2+^ induces the cleavage of filamin A via calpain activation, DU145 and PC-3 cells were treated with 3 mM CaCl2 for different time periods, and then fractionated into nuclei, crude membranes and cytosol by centrifugation. As shown in Figures 7A and 7B, we found that Ca_o_^2+^-stimulation causes a significant amount of calpain (calpain 1 and small unit of calpain 1 and 2) translocation (from cytosol to membrane as well as nucleus). The translocation leads to more co-localization of calpain and filamin A and more filamin A cleavage. These results were also confirmed by immunocytochemistry using a mouse monoclonal anti-calpain1/2 (small unit) antibody and a rabbit polyclonal anti-filamin A antibody in DU145 and PC-3 cells with or without Ca_o_^2+^-stimulation (Figure 8). The confocal images also show that Ca_o_^2+^-stimulation significantly increases the co-localization of calpain and filamin A (Figures 8F and 8L).

**Figure 9:**
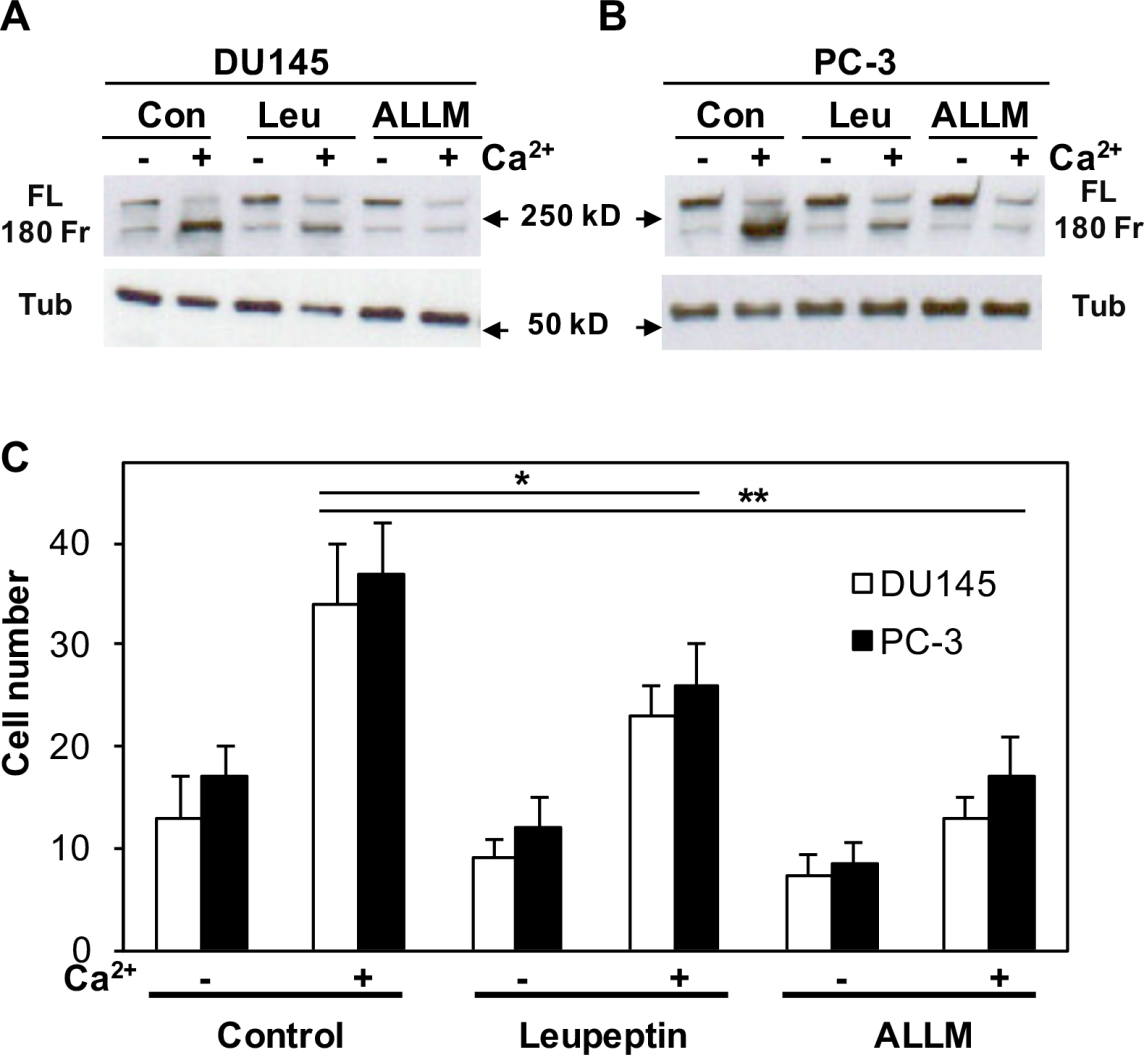
Effect of either leupeptin or ALLM on Ca_o_^2+^-induced filamin A cleavage and cell migration DU145 (**A**) and PC-3 (**B**) cells were serum deprived overnight last hour with 20 μM leupeptin (Leu) or ALLM, and then stimulated with or without 3 mM CaCl_2_ for 1 hr. The cells were lysed, and equal amounts of cellular proteins were processed for immunoblotting using the antibodies against filamin A and tubulin. Con, control; FL, full length of filamin A; 180 Fr, 180 kD fragment of filamin A; and Tub, tubulin. (**C**) The cells were seeded in 24-well plates with Transwell filters in the presence and absence of leupeptin or ALLM, and then analyzed cell migration in response to Ca_o_^2+^. The data represent means of forty fields with duplicate samples in two experiments. *P < 0.05 and **P < 0.01.

Next, we used a protease inhibitor (leupeptin) and a calpain-specific inhibitor (ALLM) to pretreat DU145 and PC-3 cells and inhibit the activation of calpain. Figures 9A and 9B illustrate that either leupeptin or ALLM can significantly block the cleavage of filamin A, although ALLM is more effective. To explore the role of calpain in the process of Ca_o_^2+^-induced cell migration, we also determined the effect of leupeptin and ALLM on DU145 and PC-3 cell migration in response to Ca_o_^2+^. As shown in Figure 9C, both leupeptin and ALLM block Ca_o_^2+^-induced filamin A cleavage, and also inhibit DU145 and PC-3 cell migration. Taken together, our results demonstrate that Ca_o_^2+^ activates CaR-mediated signaling and induces the cleavage of filamin A via calpain activation, and this cleavage promotes the migration of AR-deficient and highly metastatic prostate cancer cells.

## DISCUSSION

Many earlier studies have demonstrated that filamin A not only binds to actin but also interacts with many signaling molecules such as transmembrane receptors, nuclear receptor, signaling intermediates, and transcription factors, [9, 10, 33–39] suggesting that it may be a key signaling intermediate in a number of actin-based cellular processes. Meanwhile, filamin A is also found to be overexpressed in breast cancer [40] and AR-deficient prostate cancer cells (Figure 2A). Several laboratories have reported that filamin A can be cleaved into at least two fragments in prostate cancer cells, HeLa cells and melanoma cells [13–15, 30, 41], and that the cleaved small fragment (100 kD) can translocate to the nucleus, and directly interact with the AR, thereby modulating nuclear translocation and transcriptional action of AR in androgen-sensitive prostate cancer cells [13–15]. The AR/filamin A complex specifically regulates AR extranuclear functions leading to Rac1 activation and consequently cell motility [42]. Under hypoxic conditions, this small fragment accumulates in the cell nucleus, facilitates nuclear localization of HIF-1α, recruits HIF-1α gene promoters, and enhances HIF-1α function [30]. These studies indicate that both AR-mediated signaling and filamin A cleavage play an important role in the regulation of prostate cancer cell migration but are limited in androgen-sensitive prostate cancer cells.

Approximately 80–90% of prostate cancer is androgen-requiring for growth, survival and metastasis. Most of prostate cancer patients initially respond to ADT [43]. ADT is associated with the loss of bone mineral density and an increased risk of bone fractures for which supplementation (calcium and/or vitamin D) is commonly recommended to prostate cancer patients undergoing ADT [44, 45]. One recent study shows that prolonged androgen deprivation leads to overexpression of calpain 2 in prostate cancer progression [46]. It is very important to understand whether and how Ca_o_^2+^ regulates the migration of AR-deficient prostate cancer cells and whether Ca^2+^ or Ca^2+^-mediated signaling plays a role in the development of castrate-resistance prostate cancer (CRPC). Here, we found that Ca_o_^2+^ induces the cleavage of filamin A in AR-deficient and highly metastatic prostate cancer cell lines (DU145 and PC-3), but has no effect in androgen-sensitive prostate cancer cell line (LNCaP). Our further studies demonstrate that the cleavage of filamin A is regulated by CaR-mediated signaling and promotes the migration of AR-deficient prostate cancer cell lines. The AR is a member of steroid-thyroid-retinoid receptor (known as nuclear receptor) superfamily, while the CaR is a member of G protein-coupled receptor (GPCR) superfamily. We and others have reported that the CaR interacts with filamin A by a yeast two hybrid assay and immunoprecipitation [23, 24]. Several groups have also reported that filamin A interacts with or can be regulated by other G protein coupled receptors such as somatostatin receptor [36], dopaminereceptor [37], and opioid receptor [38]. In the present study, we further demonstrate that GPCR signaling, independent of AR, can activate the cleavage of filamin A to promote cell migration in AR- deficient and highly metastatic prostate cancer cells. Our results provide a potential rationale for a therapeutic strategy in treatment of AR-deficient prostate cancer and a mechanism of CRPC development.

Normal blood Ca^2+^ values in adults are 9.0–10.5 mg/dl or 2.25–2.75 mmol/L (1.16–1.32 mmol/L ionized Ca^2+^). The CaR can also be activated by a number of divalent cations, amino acids, and polycationic peptides, and the presence of other agonists can reduce the EC_50_ for Ca_o_^2+^ by 20–40% [47]. Our data show that the cleavage of filamin A is a dose-dependent effect in Ca_o_^2+^-treated DU145 and PC-3 cells, but not LNCaP cells. The plateau of Ca_o_^2+^-induced filamin A cleavage is at approximately 2 mM. These results suggest that Ca_o_^2+^, at physiological levels, can activate CaR-mediated signaling in AR-deficient and highly metastatic prostate cancer cells. The regulation of Ca^2+^-channels by several chemical modulators did not affect the cleavage of filamin A, while knockdown of CaR and p115RhoGEF expression attenuated filamin A cleavage. These results clearly demonstrate that Ca_o_^2+^ stimulates CaR-mediated signaling rather than Ca^2+^ entry or Ca^2+^ channels to promote the cleavage of filamin A. Recently, we and others reported that expression of the CaR is significantly up-regulated in DU145, PC-3 and C4-2B cells as compared with LNCaP cells, and prostate tumor tissue as compared with normal prostate tissue [26, 27]. Higher CaR, Gα_12_ and p115RhoGEF expression in AR-deficient and highly metastatic prostate cancer cell lines is correlated with the activation of choline kinase, cell proliferation, cell attachment, and increased metastatic behavior in response to Ca_o_^2+^ [26, 27]. It is clear that CaR-mediated signaling pathways play an important and specific role in the regulation of AR-deficient and highly metastatic prostate cancer cell migration.

Filamin A acts as a negative regulator for integrin activation by blocking talin binding to the β integrin tail [34, 35]. The cleavage of filamin A may be associated with the activation of integrin-FAK signaling and the disruption of focal adhesion [48]. Calpains are Ca^2+^-dependent proteases that can cleave filamin A at both hinge regions [7, 8, 13], but Ca^2+^ can also induce calpain translocation from the cytosol to the membrane [49, 50]. In the present study, we show that Ca_o_^2+^ induces the translocation of calpain 1 and small unit of calpain 1 and 2 from cytosol to membrane and nucleus in DU145 and PC-3 cells by cellular fractionation (Figure 7) and confocal microscopy (Figure 8). Cellular redistribution of calpain may directly be associated with the cleavage of filamin A. Treatment of DU145 and PC-3 cells with leupeptin, a naturally occurring and cell-permeable protease inhibitor that can inhibit cysteine, serine and threonine peptidases, and ALLM, a cell-permeable peptide aldehyde inhibitor of calpain 1 and 2, significantly blocked Ca_o_^2+^-induced filamin A cleavage and cell migration. The results indicate that calpains, most likely calpain 1, play a critical role in Ca_o_^2+^-induced filamin A cleavage, which leads to a remodeling of actin cytoskeleton and an increase in the migration of AR-deficient prostate cancer cells. Coincident with this, two recent reports showed that Wnt5A [41] and hypoxia [30] also activate the calpain-mediated cleavage of filamin A and increases melanoma cell motility. Recent studies indicate that calpain may be a target for limiting prostate cancer invasion [51, 52]. Our results explain the underlying mechanism and support calpain as a therapeutic target

Accumulating evidence demonstrates that cancer metastatic process is composed of a series of successive events involving interactions between tumor cells and a changing microenvironment, which drive malignant cell proliferation, migration, invasion and colonization, as well as promoting cell survival [53, 54]. Recent studies have reported that Ca_o_^2+^ stimulates the CaR leading to Rho activation, actin cytoskeleton remodeling, cell shape changes, formation of a migrating front (leading edge) and altered adhesion [18–20, 46]. Filamin A is an actin binding protein and the C-terminal portion of filamin A acts as a GTPase docking site by binding several RhoGTPases including RalA, Cdc42, Rac1, and RhoA [48, 55] which regulate cytoskeleton reorganization and cell migration. Our results show that Ca_o_^2+^ induces filamin A cleavage and promotes migration in AR-deficient and highly metastatic prostate cancer cells via a CaR-p115RhoGEF-calpain signaling pathway. Tu et al. [56] recently reported that the CaR-dependent regulation of cell-cell adhesion and keratinocyte differentiation requires Rho and filamin A, and other investigators demonstrated that Wnt5A and hypoxia activate the calpain-mediated cleavage of filamin A in melanoma cells and enhanced cell motility [30, 57]. Although we documented Ca_o_^2+^- induced filamin A cleavage, and identified part of the CaR- mediated signaling pathway in AR-deficient and highly metastatic prostate cancer cells. The complicated cellular and molecular mechanisms underlying and modulating these processes remain to be fully defined.

In summary, we show that AR-deficient and highly metastatic prostate cancer cell lines express higher levels of filamin A compared to normal prostate epithelial cells and AR-positive and less metastatic prostate cancer cells, and that Ca_o_^2+^ can induce the cleavage of filamin A in those filamin A overexpressing prostate cancer cell lines. Furthermore, Ca_o_^2+^-induced filamin A cleavage is a CaR- p115RhoGEF-calpain dependent process, and the cleavage of filamin A promotes the migration of AR- deficient and highly metastatic prostate cancer cells. Most of prostate cancer patients initially respond to ADT and the majority of them become resistant in 2–3 years and develop metastatic CRPC [43]. Understanding the CaR- mediated signaling pathway that is involved in prostate cancer cell migration could lead to new targets for drug development and therapeutic strategies for AR- deficient prostate cancer and recurrence of prostate cancer after ADT.

## MATERIALS AND METHODS

### Materials

All chemicals were purchased from Sigma Chemicals (St. Louis, MO) or Fisher Scientific (Pittsburgh, PA) unless specified otherwise. Cell culture reagents were purchased from Mediatech, Inc. (Herndon, VA). The monoclonal anti-CaR antibody used in this study was described earlier [23]. The monoclonal anti-α-tubulin antibody, and the polyclonal anti-p115 and anti-calpain 1 antibodies were obtained from Santa Cruz Biotechnology, Inc. (Santa Cruz, CA). The monoclonal anti-calpain-1/2 (small subunit) antibody was purchased from EMD Chemicals (San Diego, CA). The anti-filamin A antibodies were generated (in rabbit) against two synthetic peptides representing human filamin A in the first hinge region (AA^1755–1765^) and in the C-terminus (AA^2635–2647^) (Figure 2B). Transfection of synthetic siRNAs (negative control and CaR-specific) and establishment of stable cell lines with silenced p115 were described earlier [26]. The goat anti-mouse or rabbit Alexa Fluor 488 and Alexa Fluor 594 secondary antibodies were obtained from Molecular Probes, Inc. (Eugene, OR). The SuperSignal West Pico chemiluminescent substrate and BCA protein assay reagent were obtained from Pierce (Rockford, IL).

### Cell culture and treatment

PE, LNCaP, DU145 and PC-3 cells were grown in 6-well plates or 100 mm dishes as earlier described [26]. The lentiviral AR expressed PC-3 cells were kind gifts of Dr. John T. Isaacs (The Sidney Kimmel Comprehensive Cancer Center at Johns Hopkins University) [58]. In androgen-deprivation experiments, LNCaP cells were cultured in 10% charcoal-stripped fetal bovine serum for 3 days. The cultures were serum-deprived overnight, and then were incubated in serum-free RPMI 1640 in the presence of 3 mM CaCl_2_ for different periods of time or with different concentrations of CaCl_2_ for 60 mins. DU145 and PC-3 cells cultured in 6-well plates were transiently transfected with synthetic siRNA using Lipofectamine^TM^ 2000 reagent, and incubated at 37°C for ~ 48 hrs. The medium was replaced with serum-free RPMI 1640 for 1 hr, and the cells were treated with or without 3 mM CaCl_2_ for 1 hr. To determine the effect of different chemical regulators on Ca_o_^2+^-induced filamin A cleavage, the cells were pretreated with 100 μM tetrandrine, 10 μM FPL-64176, 25 μM SK&F96365, 20 μM leupeptin or 20 μM ALLM during last hour of serum-deprivation, and then stimulated with or without 3 μM CaCl_2_ for another 1 hr. After stimulation, the cells in 6-well plates were lysed and harvested with colorless 1× loading buffer.

### Immunocytochemistry and immunoblotting

DU145 and PC-3 cells were cultured in 100 mm dishes with two coverslips/dish for 2 days and grew to ~50–60% confluence. The cells were serum-deprived overnight, and then treated with or without 3 mM Ca_o_^2+^ for 30 mins. The cells on the coverslips were fixed with 4% paraformaldehyde in 1× PBS for 20 mins, and permeabilized with 0.1% saponin for 5 mins on ice. The coverslips were incubated with the monoclonal anti- calpain (small unit) antibody and the polyclonal anti- filamin A antibody, and then incubated with a goat anti-mouse Alexa Fluor 488 and a goat anti-rabbit Alexa Fluor 594 antibodies. The cells were viewed and photographed with a confocal microscope (Zeiss Model LSM-5 Pascal). The rest of the cells in the dishes were harvested and homogenized. The samples were centrifuged at 1000 × g for 10 mins, and the post nuclear supernatant was centrifuged at 100,000 × g for 60 mins to yield nuclei, crude membranes and cytosol.

PE, LNCaP, DU145 and PC-3 cells were lysed in colorless 1× loading buffer, and the samples were boiled for 5 mins. The lysates were centrifuged at 15,000 × g for 60 mins; cellular proteins in the supernatants and cell fractionated samples were measured using the BCA protein assay reagent with BSA as a standard. Equal amounts of cellular proteins (15 μg/lane) were subjected to SDS-PAGE, and processed for immunoblotting with the appropriate antibodies.

### Cell migration assay

LNCaP, DU145 and PC-3 cells were serum-deprived overnight, trypsinized and counted. A 0.2 ml cell suspension containing 10,000 cells was seeded into the upper chamber of Transwell filters (Costar, Cambridge, MA), in which the two chambers are separated by a polycarbonate membrane (10-μm thick and with a pore diameter of 8.0 μm). The lower chambers were filled with 0.6 ml of serum-free medium with 50 μM or 3 mM Ca_o_^2+^. The cells were allowed to migrate for 18 hrs in a 37°C incubator. Discarding the medium, the cells were washed once with 1× PBS, fixed with 4% paraformaldehyde for 20 mins, and then washed twice with 1 X PBS. The cells were stained with 0.5% crystal violet in 10% ethanol for 20 mins, and washed with water until colorless. A cotton-tipped applicator was used to remove non-migratory cells and cut the membrane from out of the chamber, and mount it with DAPI. The stained cells were photographed and counted at least ten fields per filter. The results for each experiment are presented as the mean value of two or three separate wells. For the effect of inhibitors on calpain, 20 μM leupeptin or ALLM was presented during serum deprivation and Ca_o_^2+^-stimulation periods.

### Data analysis

The data were analyzed for significance using one-way repeated measures of ANOVA followed by Tukey’s test for comparisons between the experimental groups shown in the figures.

## Abbreviations

AR: androgen receptor
Ca_o_^2+^: extracellular Ca^2+^
CaR: Ca^2+^-sensing receptor
CRPC: castrate-resistance prostate cancer
GPCR: G protein coupled receptor
siRNA: small interfering RNA.
